# Supplementary material for: TMT-Based Comparative Proteomic Analysis of the Spermatozoa of Buck (Capra hircus) and Ram (Ovis aries)
Source: Genes (Basel). 2023 Apr 25;14(5):973. doi: 10.3390/genes14050973 (PMC10218022; doi:10.3390/genes14050973)
Supplement: Supplementary file 1 [file genes-14-00973-s001.zip › Supplementary Material (Figures S1-S3).pdf]

Supplementary Material (Figures S1-S3)

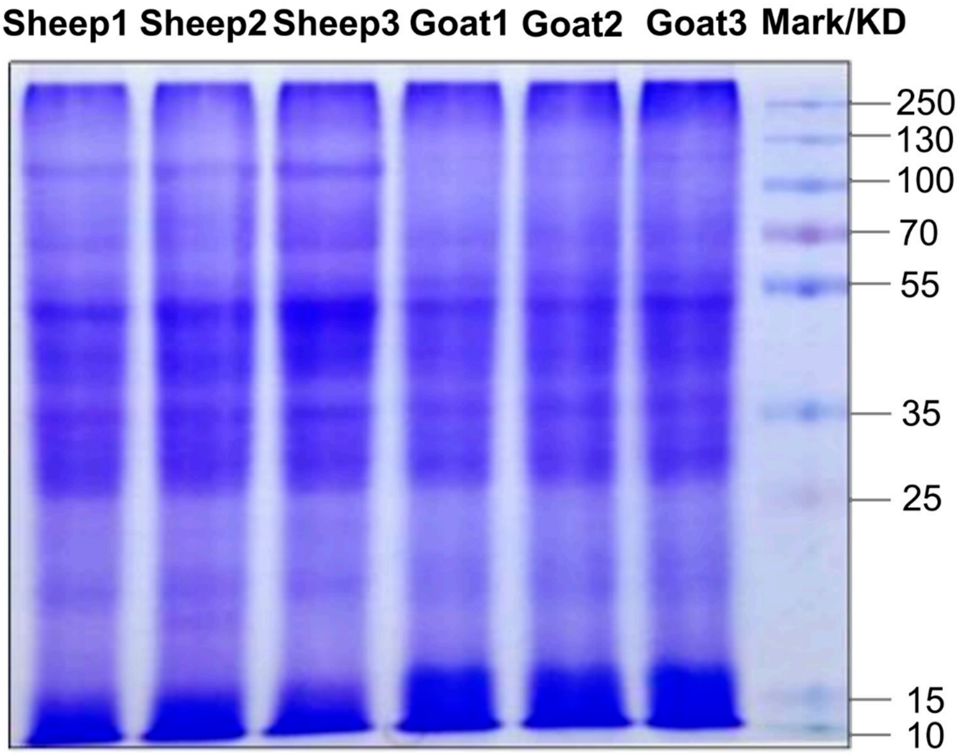

Figure S1 Electrophoresis profile of proteins extracted from spermatozoa. Mark, molecular mass protein standards; Goat1, Goat2 and Goat3, three biological replicates from goat spermatozoa; Sheep1, Sheep2 and Sheep3, three biological replicates from sheep spermatozoa.

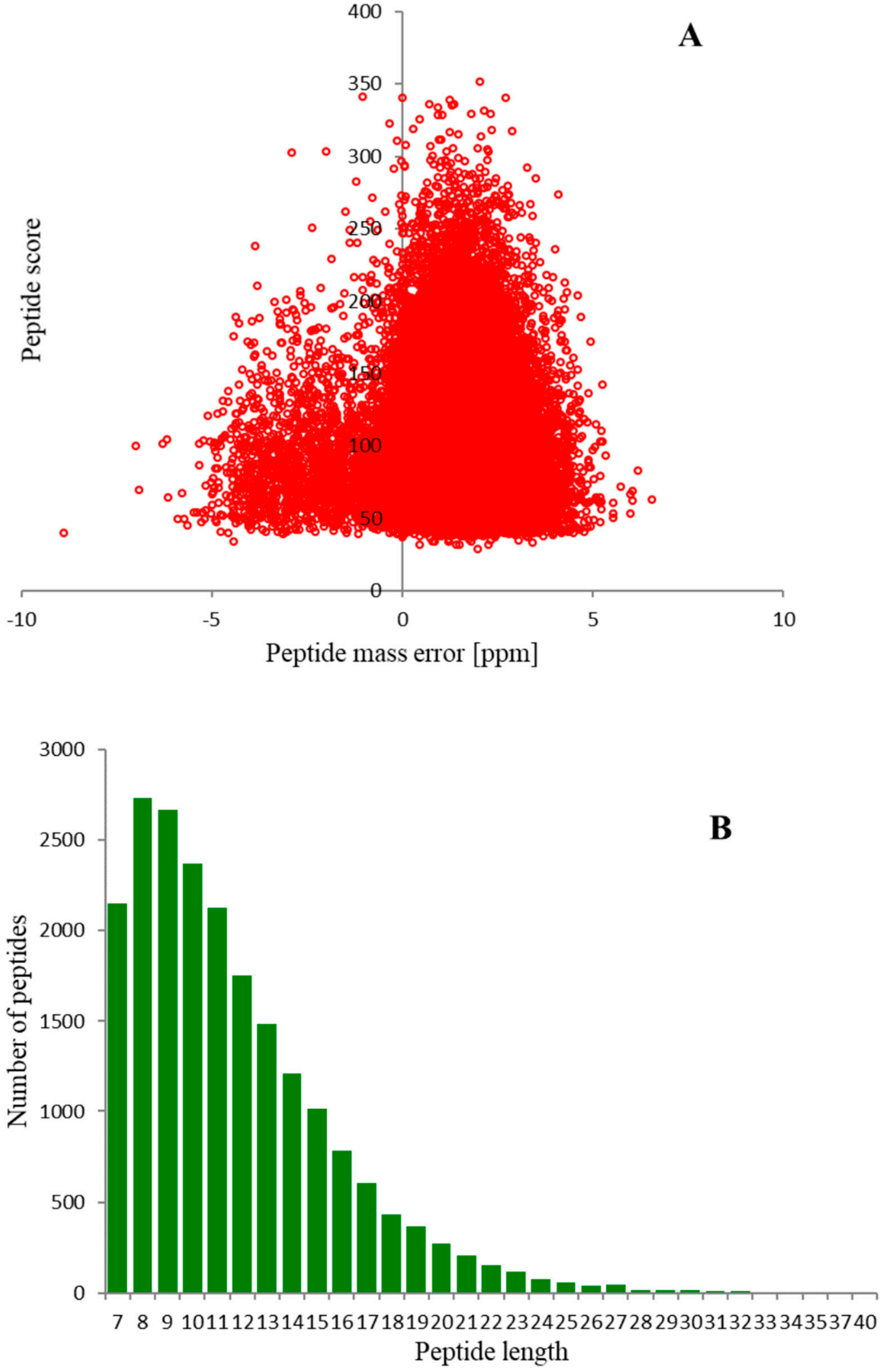

Figure S2 Peptide mass error and peptide length. (A) All the identified peptides were examined by mass error distribution. (B) Lengths of most peptides were between 8 and 20 amino acids.

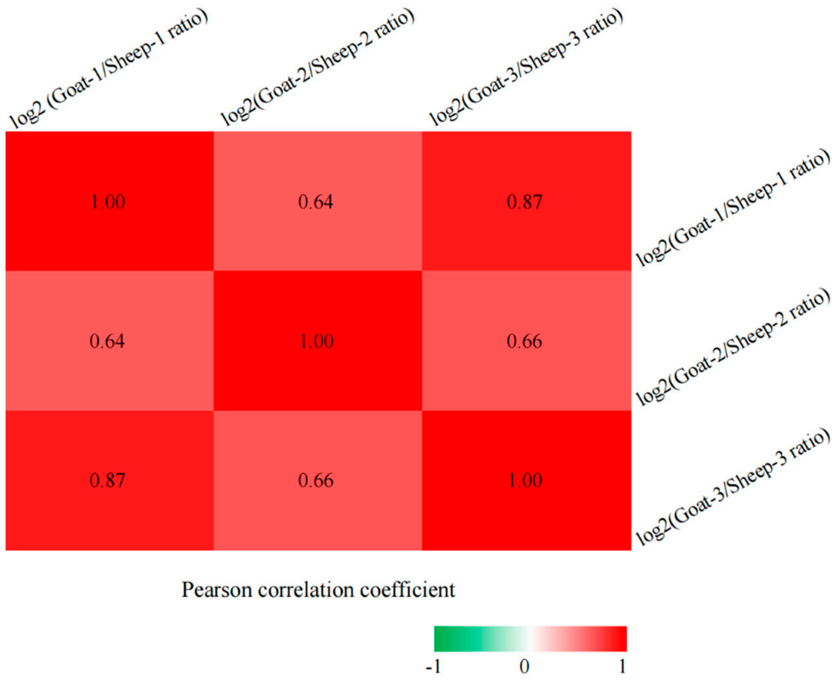

Figure S3 Pearson correlation coefficient heat map of protein quantification between two group samples.
